# Investigating Human Mitochondrial Genomes in Single Cells

**DOI:** 10.3390/genes11050534

**Published:** 2020-05-11

**Authors:** Maria Angela Diroma, Angelo Sante Varvara, Marcella Attimonelli, Graziano Pesole, Ernesto Picardi

**Affiliations:** 1 Institute of Biomembranes, Bioenergetics and Molecular Biotechnologies (IBIOM), National Research Council, Via Giovanni Amendola 118, 70126 Bari, Italy; mariangeladiroma@gmail.com (M.A.D.); graziano.pesole@uniba.it (G.P.); 2 Department of Biosciences, Biotechnology and Biopharmaceutics, University of Bari “A. Moro”, Via Orabona 4, 70125 Bari, Italy; a.varvara6@studenti.uniba.it (A.S.V.); attimonellimarcella@gmail.com (M.A.)

**Keywords:** scWGS, mtDNA, single-cell

## Abstract

Mitochondria host multiple copies of their own small circular genome that has been extensively studied to trace the evolution of the modern eukaryotic cell and discover important mutations linked to inherited diseases. Whole genome and exome sequencing have enabled the study of mtDNA in a large number of samples and experimental conditions at single nucleotide resolution, allowing the deciphering of the relationship between inherited mutations and phenotypes and the identification of acquired mtDNA mutations in classical mitochondrial diseases as well as in chronic disorders, ageing and cancer. By applying an *ad hoc* computational pipeline based on our MToolBox software, we reconstructed mtDNA genomes in single cells using whole genome and exome sequencing data obtained by different amplification methodologies (eWGA, DOP-PCR, MALBAC, MDA) as well as data from single cell Assay for Transposase Accessible Chromatin with high-throughput sequencing (scATAC-seq) in which mtDNA sequences are expected as a byproduct of the technology. We show that assembled mtDNAs, with the exception of those reconstructed by MALBAC and DOP-PCR methods, are quite uniform and suitable for genomic investigations, enabling the study of various biological processes related to cellular heterogeneity such as tumor evolution, neural somatic mosaicism and embryonic development.

## 1. Introduction

Mitochondria are subcellular organelles involved in energetic metabolism through three main sets of biochemical reactions: the tricarboxylic acid cycle (TCA cycle or Krebs cycle), the respiratory chain (RC) and the ATP synthesis machinery [1]. With few exceptions a typical eukaryotic cell has hundreds or thousands of these organelles and these numbers vary among species and tissue types depending on specific energetic needs [2]. Each mitochondrion hosts multiple copies of its own small genome, consisting of a double-stranded circular DNA molecule of 16.5 kilobase pairs that carries only 37 genes, 13 of which encode essential subunits of the oxidative phosphorylation system. Mutations occurring at mitochondrial DNA (mtDNA) can compromise the production of ATP and lead, in humans, to a variety of multisystemic disorders mainly involving high energy demanding tissues, such as skeletal muscle, the central nervous system and heart muscle [3].

mtDNA has a very high mutation rate and, compared to nuclear DNA [4], its variants can be either heteroplasmic (where both mutated and wild type mtDNA molecules co-exist within the cell) or homoplasmic (only mutated copies are present), and the phenotypic severity of mtDNA linked disorders depends on heteroplasmy levels [4,5,6,7,8,9]. 

The reconstruction of full and high quality mtDNA, as well as detecting the complete and real repertoire of its variants, is becoming a routine approach. Whole genome and exome sequencing through massive sequencing platforms such as Illumina have enabled DNA genotyping in a large number of samples and experimental conditions and improved the discovery of mtDNA variants and their quantification [10,11,12,13,14]. A few years ago, we released MToolBox, a software package implementing a fully automated pipeline for heteroplasmy quantification, annotation, and prioritization of human mtDNA variants in whole genome (WGS), whole exome (WES) and Sanger sequencing data [15]. MToolBox is currently also used to reconstruct mtDNA genomes when mtDNA is off-target in the designed sequencing library thanks to the presence in the nuclear genome of NumtS [16,17,18,19], nuclear fragments derived from mitochondrial DNA during evolution. Once the mtDNA is reconstructed, MToolBox detects the variants, quantifies heteroplasmy and assigns haplogroups according to the last Phylotree release [20], allowing the deciphering of the relationship between inherited mutations and phenotypes and the identification of acquired mtDNA mutations, not only in classical mitochondrial diseases but also in chronic diseases, aging and cancer [15,21].

However, considering that recent technological advances allow the genomic profiling of single cells at unprecedented resolution and promote the study of various biological processes related to cellular heterogeneity such as tumor evolution, neural somatic mosaicism or embryonic development [22,23], exploring mtDNA of single cells is a research topic that cannot be neglected. 

Single-cell whole genome sequencing requires an amplification step, based on the use of DNA polymerases, before its characterization because only a few picograms of DNA are present in a cell (6–7 pg for a typical human cell) (de Bourcy et al., 2014). Among current methods for single-cell Whole Genome Amplification (scWGA), the Degenerate Oligonucleotide-Primed PCR (DOP-PCR) [24] and the Multiple Displacement Amplification (MDA) [25] are commonly used. DOP-PCR yields genomes with low coverage and has proven to be accurate for detecting Copy Number Variation (CNV) [26]. MDA, instead, has high coverage but lower precision/sensitivity in CNV discovery due to amplification biases [26]. Recently, the emulsion whole-genome amplification (eWGA) method based on an improved MDA has also been released [27]. In contrast to the above WGA strategies, Multiple Annealing and Looping-Based Amplification Cycles (MALBAC) [28] is a method based on quasi-linear amplification that overcomes limitations due to the random amplification. All these methodologies are routinely used to detect and investigate naturally occurring somatic mutations, including CNVs and Single Nucleotide Variants (SNVs), in the nuclear fraction of the genome.

Single cell WGS (scWGS) experiments include mtDNA sequences that could be assembled in entire mtDNA genomes for lineage tracing or for detecting cell-specific somatic variants undetectable in bulk tissues. By applying an *ad hoc* computational pipeline based on our MToolBox software [15], we have been able to reconstruct mtDNA genomes from public scWGS data obtained by different scWGA methods (eWGA, DOP-PCR, MALBAC, MDA) that are suitable for studying biological processes related to the cellular heterogeneity in different physiological and pathological development status. Our MToolBox pipeline has also been applied to single cell Assay for Transposase Accessible Chromatin with high-throughput sequencing (scATACseq) data [29], in which mtDNA sequences, like WES data, are expected as a byproduct of the technology, yielding high quality full-length mtDNA genomes for tracing human cell lineages [29].

We aim to assess, by using the MToolBox pipeline, the relative performances in the reconstruction of mtDNA genomes of different untargeted (WGS) and targeted (WES, ATAC-seq) experimental strategies for single cell genomic analysis, adopting specific amplification protocols (e.g., DOP, MDA, eWGA, MALBAC). The overall quality of mtDNA assemblies has been evaluated based on reliability of haplogroup prediction and variant analysis.

## 2. Materials and Methods 

### 2.1. SRA Samples

We downloaded WGS and WES experiments of umbilical vein endothelial (HUVEC) cells, colon adenocarcinoma (HT-29) cell lines and related bulks from the NCBI Sequence Read Archive under the SRP052908 study accession number using the SRA Toolkit (v2.9.6) [30] prefetch utility. To speed up the download we evoked the ascp (v3.8.3.170430) function of the IBM Aspera Connect software [31], following NCBI guidelines [32]. WGS and WES experiments were preceded by DNA amplification through the following protocols eWGA, DOP-PCR, MALBAC, and MDA. Sequencing details are described in [26,27].

scATACseq data, instead, were downloaded from SRA under the accession SRP149536 (GEO ID: GSE115211) and comprise raw reads from 48 single TF-1 cells and a bulk sample. Sequencing details are described in [29].

The complete list of samples is available in Supplementary Table S1.

### 2.2. Data Pre-Processing

Sequencing data in .SRA format were converted into fastq using the fastq-dump utility from SRA Toolkit. After the FastQC (RRID:SCR_014583, v0.11.8) [33] quality check, adapters and low quality bases were trimmed using Trim Galore (RRID:SCR_011847, v0.6.1) [34], which evokes cutadapt (RRID:SCR_011841) [35], specifying an overlap of 3 nucleotides with adapter sequence required to trim a sequence (--stringency 3).

**Figure 1:**
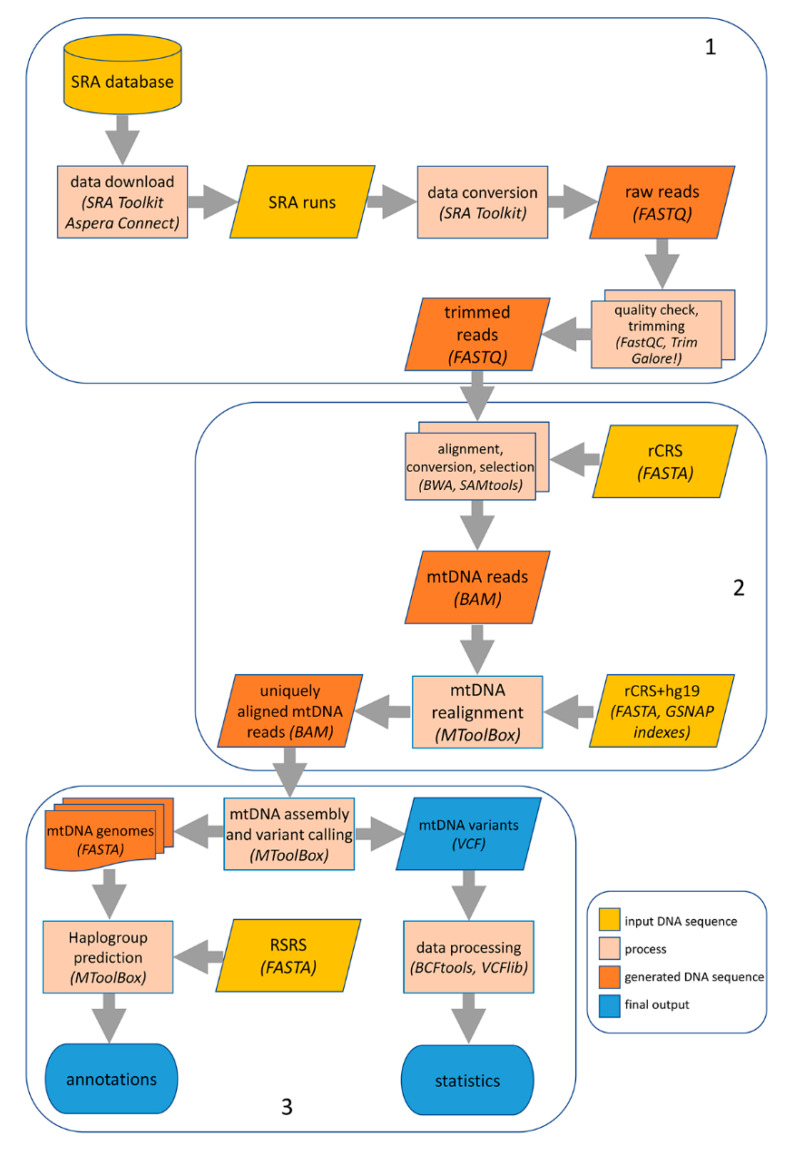
Computational pipeline for mtDNA analysis in single cell sequencing data. Our computational pipeline comprises three main steps: (**1**) download from SRA database and preprocessing of raw data from .SRA format to .FASTQ format, followed by quality check and trimming of adapters and low-quality reads; (**2**) first read alignment onto the rCRS reference sequence and subsequent realignment onto the hg19 (including rCRS) genome to obtain genuine mtDNA reads, using BWA and GSNAP (evoked by MToolBox); (**3**) mtDNA sequence assembly, variant detection and annotation by MToolBox. Further variant processing was performed with BCFtools and VCFlib.

Initially, trimmed reads were aligned using BWA (RRID:SCR_010910, v0.7.17) [36] with default parameters onto the revised Cambridge Reference Sequence, rCRS (GenBank: J01415.2) [37], to slim down the total number of reads. Then, alignments in .SAM (Sequence Alignment/Map) format were converted into the binary format (.BAM) using SAMtools (RRID:SCR_002105, v.1.3.1) [38]. The complete bioinformatics pipeline is shown in Figure 1.

### 2.3. MToolBox Analysis and Output Processing

BAM files containing reads mapped onto the mtDNA were passed to MToolBox (RRID:SCR_012112, v1.1) [15] for a complete mtDNA analysis (Figure 1), which includes several steps, from genome assembly to variant calling, annotation, up to haplogroup prediction. During these steps, MToolBox evokes external tools such as GSNAP (RRID:SCR_005483, version 2015-12-31) [39] and MUSCLE (RRID:SCR_011812, v.3.8.31) [40] for sequence alignment, and SAMtools [38] and Picard Tools (RRID:SCR_006525, v.1.98) [41] for data processing.

MToolBox allows one to choose between two different reference sequences, rCRS (GenBank: J01415.2) [37] and RSRS [42] for sequence alignment. We selected the traditional reference sequence, rCRS, for read mapping since it remains the most used in the mtDNA research community, with most databases reporting nucleotide variations against it. Most default parameters were kept, including the minimum distance from the read end required to retain an insertion/deletion during variant calling (5) and the heteroplasmy threshold for variants to be reported in the FASTA consensus sequence output (0.8). Moreover, PCR duplicates removal was enabled and the minimum threshold for quality score was set to 30. 

The resulting FASTA sequence is then used as an input to the *mt-classifier* implemented in MToolBox, which uses the aligner MUSCLE [40] and the last Phylotree build (release 17) [20] to predict the mtDNA haplogroup [43,44]. In this case, MToolBox uses RSRS as the reference sequence, representing the reconstructed root of mtDNA phylogeny [42].

One of the main outputs generated by MToolBox is a .VCF (Variant Call Format) file, reporting mutational events (mismatches, insertions, deletions) when the read depth supporting the event is ≥5 [15]. Further data processing and statistics were performed using BCFtools (RRID:SCR_005227, v.1.9) [38] stats, norm, view and fill-tags functions, and VCFlib toolkit (RRID:SCR_001231, VCFlib toolkit. Available online: https://github.com/ekg/vcflib (accessd on 02/05/2019) NOT HERE) for VCF conversion to tabular format, while BCFtools filter was used to discard low heteroplasmy variants. Annotation of identified variant sites, reported by MToolBox, was integrated by data available through the HmtVar database [45] and derived by applying the HmtNote tool [46].

The coverage of the nuclear genome was calculated using fastMitoCalc [47] for HT-29 and HUVEC cells, and SAMtools [38] and a custom python script for TF-1 cells.

## 3. Results and Discussion

### 3.1. Computational Pipeline to Characterize MtDNA Genomes in Single Cells

In contrast with bulk WGS data, single cell DNA sequencing requires an amplification step to increase the tiny amount of the input cellular DNA. ScWGA, through pure PCR or MDA, makes extensive use of DNA polymerases, leading to different technical errors such as allelic imbalance or allelic dropout, uniformity dropping and false SNVs. To capture genuine mtDNA reads and take into account potential technical biases in scWGS data, we adapted our computational pipeline to single cells WGS data, initially designed for reconstructing mtDNA genomes in WGS and WES experiments from bulk tissues [13,15] (Figure 1).

According to our methodology, raw reads from scWGS runs were pre-processed by FASTQC for evaluating the quality of each dataset and, subsequently, trimmed for removing error prone reads regions. Next, cleaned reads were subjected to a first alignment round onto the revised Cambridge Reference Sequence (rCRS) of human mtDNA genome [37] through the BWA mapper [36], in order to slim down the total number of reads per cell. The resulting pool of reads, enriched in mitochondrial sequences, was passed to our MToolBox software [15] to reconstruct the mtDNA genome and perform downstream analyses for its characterization (haplogroup assignment and variant prioritization). In MToolBox, reads were realigned using GSNAP [39] onto the complete human genome (nuclear genome and rCRS mtDNA) to filter out PCR duplicates and reads likely belonging to Nuclear mitochondrial Sequences (NumtS) originating from different portions of the mtDNA genome across the evolution [17,18,19]. Due to their similarity to mtDNA fragments, NumtS can confound the phylogenetic reconstructions [48] and affect the detection of heteroplasmic variants in studies aimed at the identification of mtDNA mutations linked to human disorders [49]. In our pipeline, NumtS were removed through two selective alignment rounds: the first one onto the mtDNA genome to collect candidate mtDNA reads and the second one onto the complete genome to discard reads aligning to both nuclear and mtDNA sequences with the same score (further details are described in the original MToolBox work [15]).

Genuine mtDNA reads were finally assembled in contigs employing a python module invoking the *mpileup* function of SAMtools [38] and further analyzed by the embedded *mt-classifier* tool to predict the most likely haplogroup according to the last update version of Phylotree [20]. This last step was performed using MUSCLE [40] and the RSRS-based Phylotree resource [20]. Haplogroups were predicted using sites covered by at least five reads and with a heteroplasmic level higher than 0.8 [15].

For each dataset, MToolBox also provided a VCF file with detected mtDNA variants including insertions, deletions and mismatches as well as the corresponding number of high quality supporting reads.

### 3.2. Reconstruction of MtDNA in Single-Cells

The above described pipeline was used to reconstruct mtDNA genomes in the SRP052908 dataset by Yusi Fu et al. [27]. It includes whole exome (WES) and whole genome (WGS) sequencing runs from HT-29 colorectal adenocarcinoma cell lines and WGS experiments only from umbilical vein endothelial cells (HUVEC). Both WES and WGS experiments were carried out using three different genome amplification protocols such as eWGA (an enhanced MDA method), DOP-PCR and MDA. In addition, WES and WGS of both cell lines were also generated using the MALBAC method based on quasi-linear amplification of DNA. For HT-29, the dataset comprises a bulk sample and nine single cells. Of these, two were sequenced with MDA, eMDA, DOP-PCR and MALBAC protocols. For HUVEC, instead, the dataset includes 10 single cells from two individuals (personal communication) and a few of them sequenced at high depth with eMDA, MDA and MALBAC protocols. In addition, for two HUVEC cells, technical replicates are also available (Supplementary Table S1).

As a result, we were able to reconstruct nearly complete mtDNA genomes with the only exception of HUVEC cells HU-eMDA10 (SRR1777289) and HU-MAL2 (SRR1777306), in which the single-end sequencing strategy was used (Supplementary Table S1). In WGS runs of HT-29 cell lines, our pipeline yielded mtDNAs with a mean coverage of 94.93% and an average per base depth of 126.81. In HUVEC cells, instead, we reconstructed mtDNAs with a mean coverage of 92.74% and an average per base depth of 3371.19. Differently from WGS, WES runs from single HT-29 cells yielded mtDNA assemblies with a lower mean coverage of 82.68% and a lower average per base depth of 27.97 (Supplementary Table S1). This finding is mostly due to a reduced number of reads in WES than in WGS and to the fact that in WES mtDNA reads are an off-target of the enrichment methodology and their abundance is strictly related to the enrichment strategy [13].

We also investigated the effect of the genome amplification strategy on the reconstruction of mtDNA genomes. While WGS from MDA, eWGA and DOP-PCR methods yielded full mtDNA genomes (mean coverage > 99%) with high depth, MToolBox on WGS MALBAC runs returned highly fragmented assemblies (>30 contigs per run) with low per base depth (11.6 on average). In contrast, only WES from eWGA and MDA yielded nearly full mtDNA genomes with an average per base depth of 39 and 27, respectively (Supplementary Table S1). 

We also applied our pipeline to scATACseq experiments from the SRP149536 study [29]. ATACseq is a sensitive method used to study chromatin accessibility in different cell types and organisms [50]. During the library preparation, the mtDNA is amplified as a byproduct of the methodology. To our aims, we selected 48 scATACseq runs from TF-1 cells and assembled full mtDNA genomes with a pretty high per base depth (243 on average) (Supplementary Table S1).

**Figure 2:**
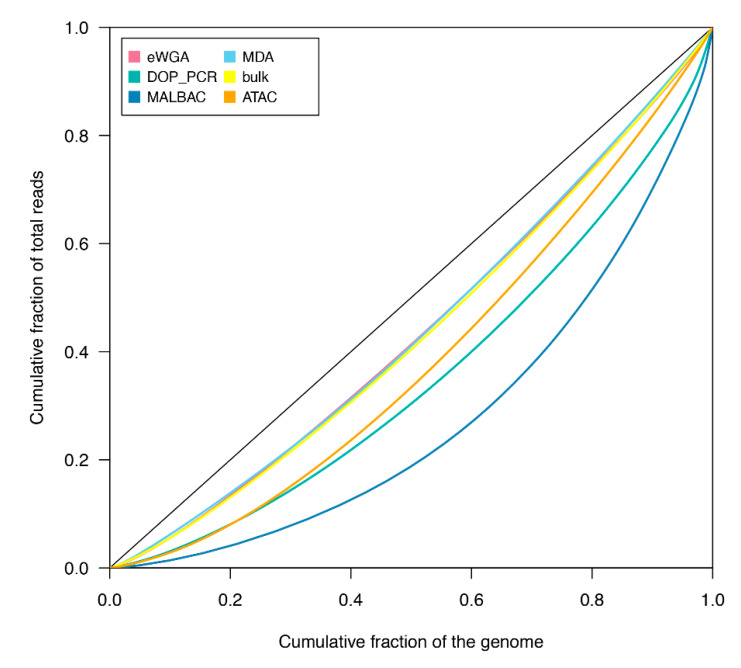
Lorenz curves of coverage uniformity for reconstructed mtDNA genomes. The cumulative fraction of sequence reads against the cumulative fraction of genome covered is shown for each amplification protocol (eWGA, DOP-PCR, MALBAC and MDA) followed by WGS in HT-29 cells, the unamplified bulk of HT-29 and scATACseq data. The diagonal line represents the ideal perfect uniform coverage. The plot was generated using the Lc function in R package *ineq*.

The cumulative fraction of sequence reads was compared with the cumulative fraction of the covered mtDNA genome in the Lorenz curve to evaluate the uniformity of reconstructed genomes (Figure 2). Since the diagonal line of the Lorenz curve represents an ideal perfect uniform coverage, observed deviations from the diagonal line indicate biases in the genome coverage. As a control, we included in our analysis WGS reads from the unamplified bulk. As shown in Figure 2, mtDNA genomes assembled from the MDA method exhibited a nearly uniform coverage relative to the unamplified bulk, with a curve quite close to the ideal diagonal line. A uniform coverage was observed also in mtDNA genomes reconstructed from eWGA and scATACseq methods. In contrast, mtDNAs from DOP-PCR and MALBAC displayed more biases and a less uniform coverage than mtDNA genomes assembled from other methodologies.

Together our results indicate that full mtDNAs can be assembled from scWGS data and amplification based strategies for genome sequencing yield quite uniform mtDNA genomes. In contrast, mtDNA genomes reconstructed using the MALBAC method are incomplete and, likewise mtDNA genomes from DOP-PCR, show an uneven coverage distribution. Full and uniform mtDNA genomes can also be assembled from scATACseq experiments that, in turn, provide a further resource and opportunity for studying mitochondrial genomics at the single cell level.

### 3.3. Quality Assessment of Reconstructed MtDNA Genomes by Haplogroup Prediction

We assessed the quality of reconstructed genomes performing the haplogroup prediction through the MToolBox program. Indeed, in addition to a VCF file with a list of single nucleotide variants and insertions/deletions (indels), it returns, after the assembly step, a consensus FASTA sequence, which is then submitted to the haplogroup prediction module (Supplementary Table S2). This module scans the mtDNA genome and the detected SNPs in order to identify variants linked to known haplogroups (Hg) according to the Phylotree (ph) classification [20]. To each predicted haplogroup, MToolBox assigns a reliability score (P_Hg), defined as the highest fraction of SNPs associated to a haplogroup (Nph) over the expected number of SNPs characterizing the haplogroup (Nph_exp) [43,44]. The higher the completeness of the genome assembly (in terms of coverage and depth), the higher the accuracy of the prediction and, thus, the possibility to unambiguously identify the haplogroup.

In all mtDNA genomes reconstructed from HT-29 cell lines, using both WGS and WES data, we predicted the haplogroup K1a1b1a to be the most likely haplogroup with an average reliability score of 97.56% (±3.45) (Supplementary Table S2). The same haplogroup was also predicted in the mtDNA genome assembled from the unamplified bulk DNA, confirming the high quality of our reconstructed sequences. In mtDNA genomes deriving from MALBAC data, the haplogroup prediction was not viable because less than 10% of these genomes were covered (Supplementary Table S2).

In HUVEC cells, we predicted two haplogroups, M7c1b2b, also detected in the unamplified bulk, and F4a1a, suggesting that these cells derive from two different donors, even though not specified in the SRA metadata but confirmed by authors (personal communication). Both haplogroups were identified with comparable accuracy, P_Hg = 97.96% and P_Hg = 96.13%, respectively (Supplementary Table S2). As in HT-29 cells, in mtDNA genome assembled from MALBAC reads the haplogroup remained unsolved because of its low coverage (6.61%).

In TF-1 cells, whose mtDNA genome was reconstructed from scATACseq data, we predicted a unique haplogroup (M7b1a1a1) with a high reliability score (P-Hg > 90%) (Supplementary Table S2).

### 3.4. Variant Detection in Reconstructed MtDNA Genomes

Despite the reduced costs of WGS and the increasing availability of ultra-depth (>10,000×) sequencing of mtDNA, deciphering the complete spectrum of mtDNA genome variants is not a straightforward process. Indeed, random and systematic errors influence and affect the accurate detection of genuine variants at very low levels. In single cells, sequencing noise is more relevant as the tiny amount of DNA per cell undergoes extensive amplification and, thus, the variant calling in mtDNA is quite challenging.

However, to demonstrate the suitability of our reconstructed mtDNAs in genomics studies, we carried out the variant calling taking into account both homoplasmic and heteroplasmic variants, employing VCF files provided by MToolBox. We initially performed a cell-to-cell variant comparison by testing different thresholds for the minimal allele frequency (from 1% to 10%) and coverage depth (from 10 to 1000) in two technical replicates (SRR1777290 vs. SRR1777291 and SRR1777287 vs SRR1777288) of HUVEC cells amplified with the eWGA method at >4000× and sequenced with the WGS protocol. (Supplementary Table S3). At a minimal allele frequency of 3% and coverage depth of 50 reads, we detected 66 and 82 nucleotide variants in the two HUVEC cells, respectively, in common between the two technical replicates corresponding to 91.7% and 89.1% of total variant detected (Supplementary Table S3). Increasing the stringency of thresholds did not improve the variant calling in terms of common nucleotide variants (Supplementary Table S3) and, thus, to recover reliable base changes we fixed the coverage depth at 50 and the minimal allele frequency at 3% for all cells sequenced at high depth. In all other cases (coverage < 1000×), the depth threshold was decreased at 30 reads. Due to amplification and sequencing noise, we never observed a perfect concordance (100% of identical nucleotide variants), even though both cells were sequenced at high depth. Allele frequencies among identical nucleotide variants were deeply correlated (>99%, Pvalue <<0.0001) and from 70% to 90% of detected changes were confirmed by the bulk (Supplementary Table S3), proving the high quality of our mtDNA genomes.

Assuming that all common variants are true positives and the remaining are false positives, at prefixed thresholds we estimated a variant calling precision (or positive prediction value, PPV) of about 90% and a prediction uncertainty of about 10%.

Then, we compared mtDNA variant profiles between HUVEC cell replicates generated using different amplification strategies. By imposing a depth of 50 reads and a minimal allele frequency of 3%, we found a concordance of 33% for MALBAC, 78% for MDA and 90% for eWGA. However, the coverage depth in replicates from the MALBAC protocol was quite unbalanced, 54× versus 211×, and a depth threshold of 50 dramatically reduced the number of shared nucleotide variants. Decreasing the depth threshold at only 10 reads, the concordance raised at 77%. These findings, therefore, show that the eWGA protocol slightly outperforms other competitor methods and suggest that the coverage depth is a critical parameter to reliably call mtDNA variants as already proved in bulk tissues [51,52,53].

The extensive DNA amplification required in the case of single cells needs more accurate exploration of the observed variants with the aim of balancing the filters implemented to minimize false detections and keep variants showing a low heteroplasmic rate.

The effect of different amplification strategies on variant calling was also checked in pairs of HT-29 cells sequenced with both WGS and WES approaches and using DOP-PCR, MALBAC, eWGA and MDA amplification protocols (Supplementary Table S4). At a depth of 30 reads and a minimum allele frequency of 3%, we obtained concordance levels higher than 87% with a very low number of private variants (detected in only one cell) (Table 1), proving again the good quality of our reconstructed genomes. However, cells sequenced using the MALBAC protocol yielded low coverage mtDNAs and a tiny number of variants, making this strategy not useful for profiling mtDNA genome variants.

Next, we compared mtDNA variant profiles from nine single HT-29 cells, whose DNA was amplified by the eWGA protocol (Supplementary Tables S4 and S5). Imposing a depth threshold of 30 reads and a minimal allele frequency of 3%, we detected 44 base variants in at least two cells (Table 2). Of these, 37 were homoplasmic or quasi- homoplasmic (with allele frequency > 95%), while the remaining seven changes were likely heteroplasmic, displaying allele frequencies from 6% to 38%.

Seventy-seven percent of all variants were confirmed by the bulk. Profiles were quite similar across cells with 82% of variants shared by more than 50% of cells, indicating high quality of our assembled genomes. The same number of HT-29 cells was also sequenced through the WES method preceded by the eWGA amplification. Here we identified 40 base changes and 34 of these (85%) were confirmed by the bulk. WES mtDNA variant profiles were less related than WGS profiles, with only 27% of changes shared by more than 50% of cells, a finding mainly due to differences in the coverage depth. Indeed, WGS yielded mtDNAs at 138x on average, while WES returned mtDNA genomes at 38x on average. Nonetheless, WGS and WES mtDNA variant profiles had a strong overlap. All 40 WES variants were shared by WGS and showed highly correlated allele frequencies (96%, pval <<0.0001). WGS exclusive variants were supported by at least two cells, even though apparently not confirmed by the bulk, suggesting that these variants could be base changes undetectable in the bulk by a population effect and not simple amplification errors.

Finally, we compared variants detected in mtDNA genomes of 48 TF-1 cells reconstructed from scATACseq data, which was enriched for mtDNA and enabled a 17-fold or greater enrichment of mtDNA compared to WES or WGS technologies [54] (Supplementary Table S5). Since mtDNA genomes were assembled at an average depth of 240x, we detected variants imposing a minimal allele frequency of 3% and at least 30 reads per site. In all 48 cells, we identified 49 base changes, including 42 homoplasmic or quasi-homoplasmic variants (allele frequency > 95%) and seven potential heteroplasmic mutations with an allele frequency ranging from 4% to 20%. Ninety percent of detected changes were confirmed by the bulk (Table 3 and Supplementary Table S5) and 42 out of 49 variants were called in more than 50% of cells, also confirming, for scATACseq data, the high quality of our mtDNA genomes and the suitability of MToolBox for mitochondrial genomics at the single cell level.

Detected variants have been further validated by using HmtVar [45] (Supplementary Table S5) as well as other relevant resources such as MitoMap [55], OMIM [56] and Clinvar [57], proving once again the efficiency of our approach.

## 4. Conclusions

Advancements in sequencing technologies and bioinformatics resources have dramatically improved mitochondrial genomics, facilitating the understanding of the major pathogenic mechanisms of mitochondrial diseases. Nonetheless, additional efforts are needed to accurately decipher the complete repertoire of mtDNA variants in a sample. This goal is particularly relevant at the single cell level because of an increased sequencing error noise. Indeed, very low quantities of DNA per cell require extensive amplification before deep sequencing. In addition, single cells should carry reliable variants, also at low heteroplasmic fraction, not detectable in the bulk by a population effect.

In the present work we have adapted our bioinformatics pipeline, based on MToolBox, to single cells WGS, WES and ATACseq data in order to reconstruct and characterize the mtDNA. Our results strongly indicate that full mtDNA genomes can be assembled at single cell levels from WGS, WES and ATACseq data. For WGS and WES runs, the amplification steps are not an issue and different protocols such as eWGA, DOP-PCR and MDA yield good and uniform mtDNA genomes. The quasi-amplification method implemented in the MALBAC protocol, instead, provides low coverage and fragmented mtDNA genomes and, consequently, it is not suitable for large scale mitochondrial genomics investigations. Reconstructed mtDNA genomes have a high quality as assessed by predicting the correct haplogroup per cell with high confidence. 

Using HT-29, HUVEC and TF-1 cells from WGS, WES and ATACseq data we show that mtDNA variants can be effectively detected in multiple cells, making our reconstructed mtDNA genomes suitable for genomics studies and for tracing clonal evolution of single cells. By fixing specific thresholds for allele frequency and depth of coverage we distinguished likely genuine changes from artifacts, especially at homoplasmic or quasi-homoplasmic variants. Results from technical replicates at high (>4000×) and low (<1000×) coverage depth indicate that mitochondrial variants can be called at the single cell level with a precision of about 90%.

However, the specificity of the detection is yet challenging and additional experimental evidence is needed to address this task. Large datasets, technical replicates and novel sequencing strategies could also be required to provide novel insights into the heteroplasmy of single cells as well as the reliable detection of linked heteroplasmic alleles.

## Figures and Tables

**Table 1:** Numbers of mtDNA variants detected in pairwise comparisons of HT-29 cells according to the following thresholds: depth of 30 reads and minimum allele frequency of 3%.

Protocol	All	Common	Hom	Het	%Common	Unique	Confirmed	%Confirmed
DOP	41	37	31	6	90.24	4	33	89.19
eWGA	44	40	37	3	90.91	4	36	90
MALBAC	3	3	2	1	100	0	2	66.67
MDA	43	38	36	2	87.37	5	36	94.74

Protocol: amplification protocol; All: all variants detected in each pair; Common: number of variants shared by each pair; Hom: number of homoplasmic variants; Het: number of heteroplasmic variants; %Common: percentage of variants shared by each pair; Unique: number of private variants; Confirmed: number of variants confirmed by the bulk; %Confirmed: percentage of variants confirmed by the bulk.

**Table 2:** Variants detected in nine HT-29 cells sequenced using the eWGA protocol according to the following thresholds: depth of 30 reads and minimum allele frequency of 3%.

Variant	inWGS	inWES	IsInBulk	mAF_WGS	mAF_WES	mAF_Bulk
m.73A > G	5	1	1	1.00	1.00	1.00
m.73A > T	5	1	1	1.00	1.00	1.00
m.114C > T	5	2	1	1.00	1.00	1.00
m.263A > G	6	3	1	1.00	1.00	1.00
m.310T > C	4	1	0	0.38	0.68	0
m.497C > T	5	2	1	0.99	0.99	1.00
m.750A > G	7	2	1	1.00	0.98	1.00
m.1189T > C	5	2	1	1.00	0.97	1.00
m.1352C > T	2	-	0	0.06	-	0
m.1413T > C	6	2	1	1.00	1.00	1.00
m.1438A > G	6	2	1	1.00	1.00	1.00
m.1811A > G	9	2	1	1.00	1.00	1.00
m.2706A > G	9	3	1	1.00	0.91	1.00
m.3480A > G	9	3	1	1.00	0.99	1.00
m.4769A > G	4	1	1	1.00	1.00	1.00
m.5591G > A	2	-	0	0.08	-	0
m.7028C > T	4	1	1	1.00	1.00	1.00
m.8860A > G *	4	1	0	1.00	1.00	1.00
m.9055G > A	9	6	1	1.00	1.00	1.00
m.9510T > C *	6	4	0	0.31	0.36	0.37
m.9698T > C	8	4	1	0.99	0.99	0.97
m.10398A > G *	9	2	0	0.99	1.00	1.00
m.10550A > G	9	3	1	0.99	1.00	1.00
m.10978A > G	7	4	1	0.99	0.98	1.00
m.11145C > A	3	-	0	0.06	-	0
m.11299T > C	9	7	1	1.00	0.99	1.00
m.11467A > G	9	5	1	1.00	1.00	1.00
m.11470A > G	9	5	1	1.00	1.00	1.00
m.11719G > A	8	3	1	1.00	1.00	1.00
m.11914G > A	7	3	1	1.00	1.00	1.00
m.12308A > G	7	2	1	1.00	1.00	1.00
m.12372G > A *	7	4	0	1.00	1.00	0.96
m.12954T > C	5	6	1	1.00	1.00	1.00
m.13831C > A *	5	5	0	0.37	0.34	0.22
m.14167C > T	7	9	1	1.00	1.00	1.00
m.14766C > T	9	4	1	1.00	0.99	1.00
m.14798T > C	8	6	1	1.00	0.99	0.96
m.15289T > C	2	-	0	0.08	-	0
m.15326A > G	9	3	1	1.00	1.00	1.00
m.15924A > G	9	3	1	1.00	1.00	1.00
m.16224T > C	7	6	1	0.99	1.00	1.00
m.16234C > T	7	6	1	1.00	1.00	1.00
m.16311T > C	8	5	1	1.00	1.00	1.00
m.16519T > C	5	1	1	1.00	0.97	1.00

Variant: type and genomic location of each variant in the format m.[POS][REF] > [ALT] (m: chrM; POS: genomic position; REF: reference base; ALT: alternative base); inWGS: number of cells sequenced by WGS in which the variant is detected; inWES: number of cells sequenced by WES in which the variant is detected; IsInBulk: a flag indicating if a variant is detected (1) or not detected (0) in the bulk; mAF_WGS: mean allele frequency in cells sequenced by WGS; mAF_WES: mean allele frequency in cells sequenced by WES; mAF_Bulk: allele frequency in the bulk (reported also for sites supported by less than 30 reads). mAF_Bulk values of 0 indicate that the site is not covered by reads. * indicates variants apparently not supported by the bulk because covered by less than 30 reads.

**Table 3:** Variants detected in 48 TF-1 cells sequenced using the ATACseq protocol according to the following thresholds: depth of 30 reads and minimum allele frequency of 3%.

Variant	# Cells	IsInBulk	mAF	mAF_Bulk
m.73A > G	43	1	1.00	1.00
m.150C > T	44	1	1.00	1.00
m.199T > C	45	1	1.00	1.00
m.263A > G	44	1	1.00	1.00
m.489T > C	47	1	1.00	0.06
m.750A > G	47	1	1.00	1.00
m.1438A > G	44	1	1.00	1.00
m.2706A > G	46	1	1.00	1.00
m.3572T > C *	9	0	0.04	1.00
m.4048G > A	43	1	1.00	0.01
m.4071C > T	40	1	1.00	1.00
m.4164A > G	34	1	1.00	1.00
m.4769A > G	1	1	1.00	1.00
m.5351A > G	28	1	0.99	1.00
m.5460G > A	47	1	0.99	1.00
m.6455C > T	47	1	1.00	1.00
m.6680T > C	43	1	1.00	1.00
m.7028C > T	38	1	1.00	1.00
m.7684T > C	42	1	1.00	1.00
m.7853G > A	46	1	1.00	1.00
m.8552T > C	43	1	1.00	1.00
m.8563A > C *	1	0	0.08	1.00
m.8684C > T *	1	0	0.18	1.00
m.8701A > G	40	1	1.00	1.00
m.8860A > G	1	1	1.00	1.00
m.9540T > C	47	1	1.00	1.00
m.9627G > A	46	1	0.18	0.02
m.9824T > C	47	1	1.00	0.99
m.10345T > C	47	1	1.00	1.00
m.10398A > G	45	1	1.00	1.00
m.10400C > T	45	1	1.00	0.99
m.10873T > C	44	1	1.00	1.00
m.11284C > T	36	1	0.08	0.99
m.11719G > A	44	1	0.98	1.00
m.12405C > T	47	1	1.00	1.00
m.12705C > T	44	1	1.00	1.00
m.12811T > C	47	1	1.00	1.00
m.12906C > T *	1	0	0.20	1.00
m.13239C > T *	1	0	0.07	1.00
m.14766C > T	39	1	1.00	1.00
m.14783T > C	42	1	1.00	1.00
m.15043G > A	47	1	1.00	1.00
m.15301G > A	46	1	1.00	1.00
m.15326A > G	47	1	1.00	0.01
m.16129G > A	46	1	0.99	1.00
m.16189T > C	39	1	1.00	0.99
m.16223C > T	46	1	1.00	1.00
m.16297T > C	47	1	1.00	0.17
m.16298T > C	47	1	1.00	1.00

Variant: type and genomic location of each variant in the format m.[POS][REF] > [ALT] (m: chrM; POS: genomic position; REF: reference base; ALT: alternative base); # Cells: number of cells in which the variant is detected; IsInBulk: a flag indicating if a variant is detected (1) or not detected (0) in the bulk; mAF: mean allele frequency; mAF_Bulk: allele frequency in the bulk (reported also for sites supported by less than 30 reads). * indicates variants apparently not supported by the bulk because covered by less than 30 reads.

## Supplementary Materials

The following are available online at https://www.mdpi.com/2073-4425/11/5/534/s1; Table S1: Summary of datasets analyzed in this study. For each sample we report: the SRA accession number (Run ID), the original sample name (Sample Name), the cell type (Cell Type), the sequencing technology (Sequencing Technology), the protocol used to amplify the DNA (Protocol), the mtDNA coverage calculated by MToolBox (mtDNA coverage), the number of contigs assembled by MToolBox (N. of contigs), the mtDNA average depth calculated by MToolBox (mtDNA average depth) and the nuclear coverage. Table S2: Haplogroup predictions. Mitochondrial haplogroups predicted by the MToolBox *mt-classifier* module. For each sample we report: the SRA accession number (Run ID), the original sample name (Sample Name), the best predicted haplogroup (Best predicted haplogroup) and the reliability score (P_Hg). Table S3: Comparison between HUVEC replicates sequenced at high depth (>4000×). The panel “SRR1777287 vs. SRR1777288” reports the variant comparison between SRR1777287 and SRR1777288 at different thresholds for coverage depth and allele frequency, while the panel “SRR1777290 vs. SRR1777291” reports the variant comparison between SRR1777290 and SRR1777291 at different thresholds for coverage depth and allele frequency. In each panel we report: the number of all variants detected in both cells (All), the number of variants in the first cell (C1), the number of variants in the second cell (C2), the number of common variants (Common), the percentage of common variants (%Common), the number of variants shared by the bulk (Inbulk), the percentage of variants found in the bulk (%BulkFound), the Pearson correlation between allele frequencies of common variants (Corr), the Pvalue associated with the Pearson correlation (pval), the minimal allele frequency tested (FreqCutoff), the minimal coverage depth tested (CovCutoff), the ID of the first cell (SC1name) and the ID of the second cell (SC2name). Table S4: HT-29 cells used for variant calling. The panel “HT-29 cell pairs” reports the HT-29 cell pairs sequenced by different amplification protocols and used for variant calling. The panel “HT-29 cells eWGA”, instead, reports the list of HT-29 cells sequenced by the eWGS protocol and used for variant calling. For each panel, we report: the run ID (Run ID), the SRA sample name (SRA_Sample), the amplification protocol (Protocol), the cell type (Cell Type), the sample name (SampleName) and the sequencing strategy (Sequencing). Table S5: Variant calling in HT-29 and TF-1 cell lines. The panel “HT-29_9_cells_WGS” reports variants detected in WGS runs from 9 HT-29 cells sequenced by the eWGA protocol. The panel “HT-29_9_cells_WES”, instead, reports variants detected in WES runs from 9 HT-29 cells sequenced by the eWGA protocol. Finally, the panel “TF1_48_cells” reports variants detected in ATACseq runs from 48 TF-1 cells. For each panel, we report: the variant, the frequency of appearance of the variant across all cells (AF), a flag indicating if a variant is present (1) or not present (0) in the bulk (IsInBulk), the mean heteroplasmic fraction (meanHF), the minimal heteroplasmic fraction observed across all cells (minHF) and the maximum heteroplasmic fraction observed across all cells (maxHF), the hyperlink to HmtVar, a flag per variant and cell indicating if a variant is present (1) or not present (0) in a given cell.
